# Pandemics, panic and prevention: Stages in the life of COVID-19 pandemic

**DOI:** 10.1177/0020764020924449

**Published:** 2020-05-02

**Authors:** Antonio Ventriglio, Cameron Watson, Dinesh Bhugra

**Affiliations:** 1 Department of Clinical and Experimental Medicine, University of Foggia, Foggia, Italy; 2 Barts NHS Foundation Trust, London, UK; 3 Institute of Psychiatry, King’s College London, London, UK

As human beings have travelled far and migrated, they have been exposed to diseases which may have been confined to their region of origin. Sometimes, they have carried these organisms and infectious agents with them and thence infecting others who may not have had immunity against these agents. Classic example of this is smallpox which was endemic to Europe and Asia, but the European invaders took it to the Americas where indigenous people had no immunity to it. Managing these and eliminating some of these have been great triumphs for modern medicine.

On occasion, these bacteria and viruses have caused untold misery leading to death as a result of pandemics. Pandemics are not new and over the millennia there have been different reasons and courses for pandemics, from measles, smallpox, influenza, bubonic plague, respiratory viruses and now COVID-19.

There is little doubt that as a result of global interconnectedness related to globalization and rapid transit of individuals and goods as well as the speed of spread and speed of misinformation, this global public health crisis for the last 3 months is nothing like the previous pandemics. As we write this, already at least one-fifth of humanity is under lockdown and more than a million people have tested positive (even with limited testing) with millions more who have had the virus. Inevitably this has caused havoc to national and global economies with factories, restaurants, public places, schools and universities shut down as the pandemic grips countries. No country is totally immune from it and countries have often taken very different approaches to manage the emergency. Of course, responses do also depend upon health care resources and socioeconomic factors.

Psychological and social factors in causation and management have been of interest. Various political figures including ministers and members of the royalty have tested positive and some have recovered.

In this brief commentary, we outline potential stages of response to the pandemic at a personal level as we observe it. In a way, these are not dissimilar to response to bereavement but are more specific to pandemics.

These stages can be seen as both individual or population responses and are fear, anxiety and panic, anger, acceptance and resolution. First stage is fear that something is happening which we do not know enough about. Fear can then be targeted at external sources, for example, conspiracy theories emerging and stereotyping of the Chinese followed to an extent that government officials in the United States started calling it China virus. All kinds of rumours about causation and treatment emerge and swirl around gaining a life of their own, and the period is accompanied by panic and anxiety. Because of fear, often people misread information. As pandemic spreads, individuals start to feel more anxious as they or their family or friends develop illness in varying forms. This panic that they themselves are vulnerable starts to hit home. Until now, the illness was in foreign parts which are often seen as odd and exotic and therefore unreal. This sense of reality that condition is now closer to home creates both panic and accompanying anxiety. Under the circumstances, the only practical thing that can be done is panic buying where whatever is available and can be bought must be bought partly because it gives people a degree of control and certainty whether they need the goods or not. This panic and anxiety can respond in a rebellious way where people may think that they know better and try to ignore government advice because it gives them some degree of control. This state of anxiety is likely to develop into anger where people lose faith in their respective governments. The salient and covert social contract fails. People have paid their taxes and dues to the government so that the governments can look after them. Once the feeling that they have been let down gets hold, it is likely to lead to social and civil unrest and then anarchy. This may be related to lack of information and transparency, misleading information or ineffectiveness of measures taken. Even if people feel that the governments have done enough, they may compare how other governments elsewhere have responded to the situation and that may make them feel better or worse. Furthermore, a sense of anger may be introjected as depressive thoughts or externalized as violence. This is perhaps the most dangerous time in the stages of response. Getting people to stay indoors or in lockdown is acceptable within limits. However, as has been seen in India, daily labourers or workers who do not have security of homes in big cities would want to return to their villages or hometowns and may create chaos and further spread of the infection. In metropolitan cities where often people live in overcrowded settings, disobeying the government instructions to go out in groups or with children can prove to be not only rebellious but also dangerous. Social isolation has its own problems in that in overcrowded houses where more than two generations are living together, it may not lead to what has been euphemistically called social distancing. Ideally, it should be called physical distancing with social and emotional closeness. In such settings, in isolation or lockdown conditions, the situation may create tensions and increase the likelihood of domestic abuse, violence, alcohol and other drug abuse or gambling. The common link between these is dealing with underlying anxiety and panic. It is quite likely that these social conditions will produce an increase not only in psychiatric conditions like anxiety and depression but also in rates of suicide. Isolation can also affect the vulnerable individuals to feel lonely and abandoned, thereby creating a vicious cycle of withdrawal and isolation. Relying on social media for information can itself feed into anxiety, anger, panic and paranoia. It is crucial that only reliable sources of information are accessed. We know that anxiety, panic and anger go hand in hand, but their presentation and resolution vary a lot. This stage will give way to acceptance of what is going on around the individual and lead to a sense of resolution.

To protect people and prevent the spread, it is critical that public mental health paradigms and measures are used. It is too early in the pandemic to see what could have been done differently and what are the lessons for the future pandemics. However, it is important to understand the stages that any given individual goes through and target preventive strategies accordingly.

It is worth emphasizing that although we have described five stages, they do tend to merge into each other. One of the few uncertain certainties is that the incubation period of the virus is variable because only in few countries, widespread testing has taken place with contact tracing. The uncertainty related to who gets it, who has sub-clinical infection and who recovers is all relatively vague thereby adding to fear, anxiety and panic. Such anxiety can be dealt with by clear transparent messages about the condition and its potential sequalae rather than vague pronouncements. Panic buying can be understood in the context of control so ensuring that food and other essential item supply chains continue undisturbed and individuals are assured about essential supplies.

Social distancing is a term much used but what is meant in reality is physical distancing, and as already mentioned, it is times like this that we need social and emotional closeness with those in families, our friends, peers and colleagues. Words have meanings and we need to be clear what we mean by them. In egocentric societies, people are often socially distant, so this instruction reflects that context. Furthermore, clear messages with clear language about what is needed to prevent spread of the virus are critical. At the same time, policymakers need to be clear about the resolution stage as to how this will occur and what steps will be needed. Sharing data across countries in a transparent manner irrespective of different health care systems and not relying on rumours or false information are important at all levels with policymakers, health care professionals and members of the public. Stages of pandemic can only guide us so far. The governments need to step up to protect their populations and people in a non-threatening, non-panicky manner to ensure safety of all individuals.

